# 2-(5,6-Di­hydro­benzimidazolo[1,2-*c*]quinazolin-6-yl)aniline methanol solvate

**DOI:** 10.1107/S1600536809021886

**Published:** 2009-06-13

**Authors:** Irvin Booysen, Thomas I. A. Gerber, Eric Hosten, Peter Mayer

**Affiliations:** a Department of Chemistry, Nelson Mandela Metropolitan University, 6031 Port Elizabeth, South Africa; b Department of Chemistry, Ludiwig-Maximilians University, D-81377 München, Germany

## Abstract

In the structure of the title compound, C_20_H_16_N_4_·CH_4_O, the aniline ring forms dihedral angles of 89.9 (2) and 85.4 (2)° with the benzimidazole and benzene rings, respectively. The orientation of the aniline ring is mainly determined by strong hydrogen bonds between the amino group and the non-fused quinazoline N atom. Inter­molecular hydrogen bonds of the N—H⋯N—H⋯N type along [010] and the N—H⋯O—H⋯N type along [100] are formed, resulting in *C*_2_^2^(4) and *C* ^2^_2_(10) descriptors, respectively, on a binary level of graph-set analysis. There are C—H⋯π contacts with H⋯π distances of 2.44 Å; however, no π-stacking is observed.

## Related literature

For the synthesis of quinazolines, see: Kubicova *et al.* (2003 ▶); Niementowski (1895 ▶). For the conformation, see: Cuny *et al.* (1980 ▶); Williamson (1957 ▶).
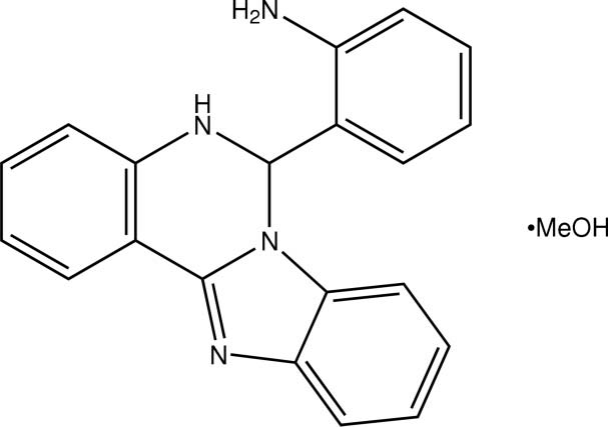


## Experimental

### 

#### Crystal data

C_20_H_16_N_4_·CH_4_O
*M* *_r_* = 344.41
Monoclinic, 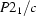

*a* = 9.3703 (2) Å
*b* = 5.1728 (1) Å
*c* = 35.5169 (9) Å
β = 91.3908 (14)°
*V* = 1721.02 (7) Å^3^
*Z* = 4
Mo *K*α radiation
μ = 0.09 mm^−1^
*T* = 200 K
0.23 × 0.06 × 0.05 mm

#### Data collection

Nonius KappaCCD diffractometer
Absorption correction: none
9302 measured reflections
3136 independent reflections
2089 reflections with *I* > 2σ(*I*)
*R*_int_ = 0.051

#### Refinement

*R*[*F* ^2^ > 2σ(*F* ^2^)] = 0.048
*wR*(*F* ^2^) = 0.123
*S* = 1.08
3136 reflections
253 parameters
H atoms treated by a mixture of independent and constrained refinement
Δρ_max_ = 0.19 e Å^−3^
Δρ_min_ = −0.20 e Å^−3^

### 

Data collection: *COLLECT* (Nonius, 2004 ▶); cell refinement: *SCALEPACK* (Otwinowski & Minor, 1997 ▶); data reduction: *DENZO* (Otwinowski & Minor, 1997 ▶) and *SCALEPACK*; program(s) used to solve structure: *SIR97* (Altomare *et al.*, 1999 ▶); program(s) used to refine structure: *SHELXL97* (Sheldrick, 2008 ▶); molecular graphics: *ORTEP-3 for Windows* (Farrugia, 1997 ▶) and *Mercury* (Macrae *et al.*, 2006 ▶); software used to prepare material for publication: *PARST* (Nardelli, 1995 ▶), *publCIF* (Westrip, 2008 ▶) and *WinGX* (Farrugia, 1999 ▶).

## Supplementary Material

Crystal structure: contains datablocks I, global. DOI: 10.1107/S1600536809021886/bg2264sup1.cif

Structure factors: contains datablocks I. DOI: 10.1107/S1600536809021886/bg2264Isup2.hkl

Additional supplementary materials:  crystallographic information; 3D view; checkCIF report

## Figures and Tables

**Table 1:** Hydrogen-bond geometry (Å, °)

*D*—H⋯*A*	*D*—H	H⋯*A*	*D*⋯*A*	*D*—H⋯*A*
N3—H3*a*⋯N4^i^	0.93 (2)	2.13 (2)	3.058 (3)	174.5 (18)
N4—H4*a*⋯O1^ii^	0.95 (2)	2.00 (3)	2.946 (3)	174.4 (19)
N4—H4*b*⋯N3	0.86 (2)	2.35 (2)	3.006 (3)	133.8 (19)
O1—H1⋯N1	0.95 (3)	1.88 (3)	2.814 (2)	167 (3)
C14—H14⋯*Cg*^i^	1.00	2.44	3.408 (2)	162

Symmetry codes: (i) 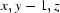; (ii) 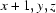. *Cg* is the centroid of the C1–C6 ring.

**Fig. 1.**
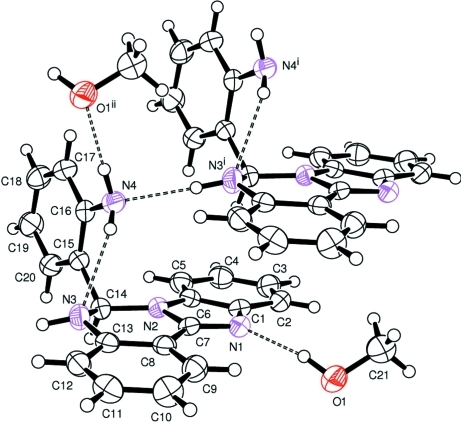
The molecular structure of the title compound (anisotropic displacement ellipsoids drawn at the 50% probability level). Hydrogen bonds determining the conformational arrangement of the analine rings are also shown. Symmetry codes: (i): x, y+1, z; (ii) x+1, y, z.

## supplementary crystallographic information

### Comment

In the present work the structure of 2-(2,3,5,6-tetrahydrobenzimidazo[1,2-*c*]quinazolin-5-yl)benzenamine (Figure 1) has been determined to explore its suitability as a bidentate ligand for various metal ions. In the structure the quinazoline ring adopts a chair conformation: atoms C7, C8, C13, N2 and N3 are coplanar, with atom C14 departing from the plane by 0.2391 Å . The orientation of the aniline ring is mainly determined by a series of hydrogen-bonds between NH_2_ and NH groups, and between the phenylamino group and the quinazoline nitrogen (Figure 1 and Table 1). This ring makes dihedral angles of 89.9 (2)° and 85.4 (2)° with the benzimidazole and phenyl rings respectively. The ligand bond distances and angles show that N1—C7 is a localized double bond [1.321 (3) Å], with N2—C7 a single bond at 1.383 (3) Å. The N3—C14 bond length is 1.459 (3) Å, and the N3—C14—N2 bond angle [108.14 (17)°] illustrates the *sp*^3^ hybridization of C14. All the other bond lengths and angles in the molecule are normal.

Intermolecular hydrogen bonds of the N–H···N–H···N type along [010] and N–H···O–H···N along [100] are formed (Table 1) resulting in a C^2^_2_(4) descriptor and a C^2^_2_(10) descriptor on a binary level of graph set analysis, respectively. C–H···π contacts with H···*Cg* distances of 2.44 Å are presnt in the structure (*Cg* is the centre of gravity of ring C1 – C6); however, no π-stacking is observed.

### Experimental

All chemicals used (reagent grade) were commercially available. A mass of 1.22 g (0.010 mol) of 2-aminobenzaldehyde was dissolved in methanol (50 cm^3^), and 2.09 g (0.010 mol) of 2-(2-aminophenyl)-1-benzimidazole was added with stirring. The mixture was heated under reflux for 2 h, then cooled to room temperature and filtered. The volume of the solution was reduced to ~ 10 cm^3^, and left to evaporate slowly at room temperature. After 2 days 2.48 g (72%) of colourless crystals, with the formulation C_20_H_16_N_4_.CH_4_O and suitable for X-ray analysis, were collected. *M*.p. 211°C. ^1^H NMR (300 MHz, d_6_-DMSO): 7.97 (1*H*,d), 7.62 (1*H*, d), 7.27 (1*H*, d), 7.25 (1*H*,t), 7.13 (2*H*, t), 6.97–7.02 (2*H*, m), 6.94 (1*H*, t), 6.86 (1*H*, t), 6.78 (1*H*, d), 6.61 (1*H*, d), 6.54(1*H*, t), 5.45 (2*H*, s), 3.18 (3*H*, s).

### Refinement

All H atoms bonded to C atoms were calculated in idealized position and refined as riding on their parent atoms with *U*_iso_(H) values of 1.2 *U*_eq_(C). All H atoms bonded to N and O atoms were refined freely with individual *U*_iso_(H) values.

### Crystal data

**Table:**

C_20_H_16_N_4_·CH_4_O	*F*(000) = 728
*M**_r_* = 344.41	*D*_x_ = 1.329 (1) Mg m^−^^3^
Monoclinic, *P*2_1_/*c*	Melting point: 484 K
Hall symbol: -P 2ybc	Mo *K*α radiation, λ = 0.71073 Å
*a* = 9.3703 (2) Å	Cell parameters from 41046 reflections
*b* = 5.1728 (1) Å	θ = 3.1–25.4°
*c* = 35.5169 (9) Å	µ = 0.09 mm^−^^1^
β = 91.3908 (14)°	*T* = 200 K
*V* = 1721.02 (7) Å^3^	Rod, yellow
*Z* = 4	0.23 × 0.06 × 0.05 mm

### Data collection

**Table:**

Nonius KappaCCD diffractometer	2089 reflections with *I* > 2σ(*I*)
Radiation source: rotating anode	*R*_int_ = 0.051
MONTEL, graded multilayered X-ray optics	θ_max_ = 25.4°, θ_min_ = 3.2°
Detector resolution: 9 pixels mm^-1^	*h* = −11→11
CCD; rotation images, φ and ω scans	*k* = −5→6
9302 measured reflections	*l* = −42→42
3136 independent reflections	

### Refinement

**Table:**

Refinement on *F*^2^	Secondary atom site location: difference Fourier map
Least-squares matrix: full	Hydrogen site location: inferred from neighbouring sites
*R*[*F*^2^ > 2σ(*F*^2^)] = 0.048	H atoms treated by a mixture of independent and constrained refinement
*wR*(*F*^2^) = 0.123	*w* = 1/[σ^2^(*F*_o_^2^) + (0.0417*P*)^2^ + 0.5855*P*] where *P* = (*F*_o_^2^ + 2*F*_c_^2^)/3
*S* = 1.08	(Δ/σ)_max_ < 0.001
3136 reflections	Δρ_max_ = 0.19 e Å^−^^3^
253 parameters	Δρ_min_ = −0.19 e Å^−^^3^
0 restraints	Extinction correction: *SHELXL97* (Sheldrick, 2008)
Primary atom site location: structure-invariant direct methods	Extinction coefficient: 0.0138 (19)

### Special details

**Table:**

Refinement. N- and O-bonded H: All H-atom parameters refined C-bonded H: H-atom parameters constrained

### Fractional atomic coordinates and isotropic or equivalent isotropic displacement parameters (Å^2^)

**Table:**

	*x*	*y*	*z*	*U*_iso_*/*U*_eq_	
O1	−0.05660 (18)	0.5134 (3)	0.32314 (5)	0.0506 (5)	
H1	0.038 (3)	0.482 (5)	0.3324 (8)	0.087 (10)*	
N1	0.20470 (18)	0.3599 (3)	0.35685 (5)	0.0349 (5)	
N2	0.38507 (17)	0.1319 (3)	0.38325 (5)	0.0321 (5)	
N3	0.5138 (2)	−0.1946 (4)	0.35148 (5)	0.0407 (5)	
H3a	0.564 (2)	−0.350 (5)	0.3544 (6)	0.043 (7)*	
N4	0.6823 (2)	0.2995 (4)	0.35560 (6)	0.0381 (5)	
H4a	0.764 (3)	0.364 (4)	0.3436 (6)	0.051 (7)*	
H4b	0.635 (2)	0.195 (4)	0.3412 (6)	0.040 (7)*	
C1	0.2283 (2)	0.4419 (4)	0.39363 (6)	0.0329 (5)	
C2	0.1591 (2)	0.6363 (4)	0.41352 (6)	0.0394 (6)	
H2	0.0863	0.7387	0.4019	0.047*	
C3	0.2005 (2)	0.6737 (5)	0.45063 (7)	0.0437 (6)	
H3	0.1545	0.8037	0.4648	0.052*	
C4	0.3083 (3)	0.5254 (5)	0.46794 (6)	0.0443 (6)	
H4	0.3332	0.5557	0.4937	0.053*	
C5	0.3792 (2)	0.3361 (4)	0.44846 (6)	0.0393 (6)	
H5	0.4528	0.2360	0.4601	0.047*	
C6	0.3383 (2)	0.2981 (4)	0.41099 (6)	0.0317 (5)	
C7	0.2981 (2)	0.1726 (4)	0.35178 (6)	0.0307 (5)	
C8	0.3146 (2)	0.0110 (4)	0.31914 (6)	0.0321 (5)	
C9	0.2270 (2)	0.0334 (5)	0.28675 (6)	0.0410 (6)	
H9	0.1572	0.1664	0.2853	0.049*	
C10	0.2408 (3)	−0.1347 (5)	0.25705 (6)	0.0475 (7)	
H10	0.1800	−0.1208	0.2354	0.057*	
C11	0.3452 (3)	−0.3251 (5)	0.25931 (6)	0.0452 (6)	
H11	0.3552	−0.4419	0.2389	0.054*	
C12	0.4343 (2)	−0.3483 (4)	0.29040 (6)	0.0393 (6)	
H12	0.5060	−0.4781	0.2912	0.047*	
C13	0.4193 (2)	−0.1808 (4)	0.32092 (6)	0.0333 (5)	
C14	0.4874 (2)	−0.0827 (4)	0.38838 (6)	0.0342 (5)	
H14	0.4431	−0.2178	0.4045	0.041*	
C15	0.6265 (2)	0.0014 (4)	0.40688 (6)	0.0322 (5)	
C16	0.7166 (2)	0.1831 (4)	0.39045 (6)	0.0337 (5)	
C17	0.8413 (2)	0.2541 (5)	0.40991 (7)	0.0423 (6)	
H17	0.9013	0.3825	0.3996	0.051*	
C18	0.8792 (3)	0.1423 (5)	0.44374 (7)	0.0478 (7)	
H18	0.9647	0.1948	0.4565	0.057*	
C19	0.7947 (3)	−0.0451 (5)	0.45936 (7)	0.0469 (6)	
H19	0.8225	−0.1265	0.4824	0.056*	
C20	0.6682 (2)	−0.1127 (4)	0.44085 (6)	0.0408 (6)	
H20	0.6086	−0.2400	0.4516	0.049*	
C21	−0.0816 (3)	0.7792 (5)	0.32160 (8)	0.0677 (8)	
H21A	−0.0188	0.8671	0.3400	0.102*	
H21B	−0.1814	0.8141	0.3275	0.102*	
H21C	−0.0623	0.8430	0.2962	0.102*	

### Atomic displacement parameters (Å^2^)

**Table:**

	*U*^11^	*U*^22^	*U*^33^	*U*^12^	*U*^13^	*U*^23^
O1	0.0392 (11)	0.0532 (12)	0.0589 (11)	0.0000 (9)	−0.0085 (8)	0.0070 (9)
N1	0.0293 (10)	0.0331 (11)	0.0421 (12)	0.0012 (9)	−0.0009 (8)	−0.0012 (9)
N2	0.0292 (10)	0.0293 (10)	0.0378 (11)	0.0002 (8)	−0.0033 (8)	−0.0017 (8)
N3	0.0379 (12)	0.0347 (12)	0.0491 (13)	0.0077 (10)	−0.0095 (9)	−0.0094 (9)
N4	0.0321 (12)	0.0386 (12)	0.0435 (13)	−0.0024 (10)	0.0001 (10)	0.0054 (10)
C1	0.0268 (12)	0.0324 (13)	0.0395 (13)	−0.0056 (10)	0.0006 (10)	0.0001 (10)
C2	0.0307 (13)	0.0357 (14)	0.0519 (15)	0.0003 (11)	−0.0005 (11)	−0.0032 (11)
C3	0.0395 (14)	0.0424 (15)	0.0494 (15)	−0.0006 (12)	0.0058 (11)	−0.0098 (12)
C4	0.0479 (15)	0.0436 (15)	0.0415 (14)	−0.0049 (13)	0.0014 (11)	−0.0057 (12)
C5	0.0403 (14)	0.0372 (14)	0.0401 (14)	−0.0034 (11)	−0.0028 (11)	−0.0009 (11)
C6	0.0306 (12)	0.0249 (12)	0.0397 (13)	−0.0049 (10)	0.0021 (10)	−0.0008 (10)
C7	0.0241 (12)	0.0301 (13)	0.0379 (13)	−0.0050 (10)	−0.0010 (9)	0.0018 (10)
C8	0.0302 (12)	0.0301 (13)	0.0360 (13)	−0.0063 (10)	0.0022 (10)	0.0023 (10)
C9	0.0403 (14)	0.0437 (15)	0.0388 (14)	0.0016 (11)	−0.0015 (11)	0.0033 (11)
C10	0.0545 (17)	0.0534 (17)	0.0344 (14)	0.0004 (13)	−0.0033 (11)	−0.0003 (12)
C11	0.0562 (16)	0.0419 (15)	0.0379 (14)	−0.0036 (13)	0.0077 (12)	−0.0035 (11)
C12	0.0425 (14)	0.0323 (14)	0.0433 (14)	0.0001 (11)	0.0058 (11)	0.0002 (11)
C13	0.0327 (13)	0.0290 (13)	0.0382 (13)	−0.0061 (10)	0.0016 (10)	0.0028 (10)
C14	0.0314 (13)	0.0280 (12)	0.0432 (13)	0.0016 (10)	−0.0025 (10)	0.0012 (10)
C15	0.0306 (12)	0.0286 (12)	0.0372 (13)	0.0003 (10)	−0.0019 (9)	−0.0004 (10)
C16	0.0302 (12)	0.0287 (13)	0.0421 (13)	0.0040 (10)	−0.0001 (10)	−0.0001 (10)
C17	0.0317 (13)	0.0411 (14)	0.0539 (15)	−0.0051 (11)	−0.0012 (11)	0.0024 (12)
C18	0.0367 (14)	0.0506 (16)	0.0554 (16)	−0.0008 (12)	−0.0142 (12)	−0.0053 (13)
C19	0.0464 (15)	0.0485 (16)	0.0451 (14)	0.0009 (13)	−0.0114 (12)	0.0031 (12)
C20	0.0363 (13)	0.0398 (14)	0.0460 (14)	−0.0013 (11)	−0.0025 (11)	0.0054 (11)
C21	0.0489 (17)	0.055 (2)	0.100 (2)	−0.0015 (14)	0.0074 (15)	0.0046 (16)

### Geometric parameters (Å, °)

**Table:**

O1—C21	1.395 (3)	C8—C9	1.402 (3)
O1—H1	0.95 (3)	C9—C10	1.376 (3)
N1—C7	1.320 (3)	C9—H9	0.9500
N1—C1	1.386 (3)	C10—C11	1.389 (3)
N2—C7	1.384 (3)	C10—H10	0.9500
N2—C6	1.387 (3)	C11—C12	1.373 (3)
N2—C14	1.475 (3)	C11—H11	0.9500
N3—C13	1.386 (3)	C12—C13	1.398 (3)
N3—C14	1.459 (3)	C12—H12	0.9500
N3—H3a	0.93 (2)	C14—C15	1.509 (3)
N4—C16	1.406 (3)	C14—H14	1.0000
N4—H4a	0.95 (2)	C15—C20	1.391 (3)
N4—H4b	0.86 (2)	C15—C16	1.400 (3)
C1—C2	1.397 (3)	C16—C17	1.393 (3)
C1—C6	1.402 (3)	C17—C18	1.372 (3)
C2—C3	1.379 (3)	C17—H17	0.9500
C2—H2	0.9500	C18—C19	1.377 (3)
C3—C4	1.399 (3)	C18—H18	0.9500
C3—H3	0.9500	C19—C20	1.386 (3)
C4—C5	1.379 (3)	C19—H19	0.9500
C4—H4	0.9500	C20—H20	0.9500
C5—C6	1.390 (3)	C21—H21A	0.9800
C5—H5	0.9500	C21—H21B	0.9800
C7—C8	1.441 (3)	C21—H21C	0.9800
C8—C13	1.396 (3)		
			
C21—O1—H1	109.6 (18)	C11—C10—H10	120.6
C7—N1—C1	105.19 (17)	C12—C11—C10	121.5 (2)
C7—N2—C6	106.81 (17)	C12—C11—H11	119.3
C7—N2—C14	125.65 (17)	C10—C11—H11	119.3
C6—N2—C14	126.49 (17)	C11—C12—C13	120.0 (2)
C13—N3—C14	124.33 (19)	C11—C12—H12	120.0
C13—N3—H3a	116.1 (13)	C13—C12—H12	120.0
C14—N3—H3a	109.7 (13)	N3—C13—C8	120.48 (19)
C16—N4—H4a	112.2 (13)	N3—C13—C12	120.1 (2)
C16—N4—H4b	111.0 (15)	C8—C13—C12	119.29 (19)
H4a—N4—H4b	111 (2)	N3—C14—N2	108.15 (16)
N1—C1—C2	129.15 (19)	N3—C14—C15	110.02 (17)
N1—C1—C6	110.53 (18)	N2—C14—C15	112.88 (17)
C2—C1—C6	120.3 (2)	N3—C14—H14	108.6
C3—C2—C1	117.5 (2)	N2—C14—H14	108.6
C3—C2—H2	121.3	C15—C14—H14	108.6
C1—C2—H2	121.3	C20—C15—C16	119.16 (19)
C2—C3—C4	121.8 (2)	C20—C15—C14	118.47 (19)
C2—C3—H3	119.1	C16—C15—C14	122.33 (19)
C4—C3—H3	119.1	C17—C16—C15	118.5 (2)
C5—C4—C3	121.4 (2)	C17—C16—N4	119.7 (2)
C5—C4—H4	119.3	C15—C16—N4	121.82 (19)
C3—C4—H4	119.3	C18—C17—C16	121.3 (2)
C4—C5—C6	117.1 (2)	C18—C17—H17	119.3
C4—C5—H5	121.4	C16—C17—H17	119.3
C6—C5—H5	121.4	C17—C18—C19	120.7 (2)
N2—C6—C5	133.1 (2)	C17—C18—H18	119.7
N2—C6—C1	104.99 (17)	C19—C18—H18	119.7
C5—C6—C1	121.9 (2)	C18—C19—C20	118.7 (2)
N1—C7—N2	112.39 (18)	C18—C19—H19	120.7
N1—C7—C8	128.31 (19)	C20—C19—H19	120.7
N2—C7—C8	119.27 (19)	C19—C20—C15	121.5 (2)
C13—C8—C9	119.5 (2)	C19—C20—H20	119.2
C13—C8—C7	117.73 (18)	C15—C20—H20	119.2
C9—C8—C7	122.7 (2)	O1—C21—H21A	109.5
C10—C9—C8	120.9 (2)	O1—C21—H21B	109.5
C10—C9—H9	119.6	H21A—C21—H21B	109.5
C8—C9—H9	119.6	O1—C21—H21C	109.5
C9—C10—C11	118.9 (2)	H21A—C21—H21C	109.5
C9—C10—H10	120.6	H21B—C21—H21C	109.5
			
C7—N1—C1—C2	179.8 (2)	C10—C11—C12—C13	1.1 (3)
C7—N1—C1—C6	−0.4 (2)	C14—N3—C13—C8	21.6 (3)
N1—C1—C2—C3	177.9 (2)	C14—N3—C13—C12	−162.73 (19)
C6—C1—C2—C3	−1.9 (3)	C9—C8—C13—N3	175.3 (2)
C1—C2—C3—C4	0.5 (3)	C7—C8—C13—N3	−7.0 (3)
C2—C3—C4—C5	0.6 (4)	C9—C8—C13—C12	−0.4 (3)
C3—C4—C5—C6	−0.4 (3)	C7—C8—C13—C12	177.30 (18)
C7—N2—C6—C5	176.7 (2)	C11—C12—C13—N3	−176.6 (2)
C14—N2—C6—C5	8.0 (4)	C11—C12—C13—C8	−0.8 (3)
C7—N2—C6—C1	−3.1 (2)	C13—N3—C14—N2	−24.8 (3)
C14—N2—C6—C1	−171.81 (18)	C13—N3—C14—C15	−148.5 (2)
C4—C5—C6—N2	179.3 (2)	C7—N2—C14—N3	17.3 (3)
C4—C5—C6—C1	−1.0 (3)	C6—N2—C14—N3	−176.03 (18)
N1—C1—C6—N2	2.2 (2)	C7—N2—C14—C15	139.21 (19)
C2—C1—C6—N2	−178.01 (19)	C6—N2—C14—C15	−54.1 (3)
N1—C1—C6—C5	−177.61 (18)	N3—C14—C15—C20	−118.8 (2)
C2—C1—C6—C5	2.2 (3)	N2—C14—C15—C20	120.3 (2)
C1—N1—C7—N2	−1.7 (2)	N3—C14—C15—C16	59.1 (3)
C1—N1—C7—C8	176.36 (19)	N2—C14—C15—C16	−61.8 (3)
C6—N2—C7—N1	3.1 (2)	C20—C15—C16—C17	−3.7 (3)
C14—N2—C7—N1	171.95 (18)	C14—C15—C16—C17	178.5 (2)
C6—N2—C7—C8	−175.15 (17)	C20—C15—C16—N4	178.1 (2)
C14—N2—C7—C8	−6.3 (3)	C14—C15—C16—N4	0.3 (3)
N1—C7—C8—C13	−178.1 (2)	C15—C16—C17—C18	2.6 (3)
N2—C7—C8—C13	−0.2 (3)	N4—C16—C17—C18	−179.1 (2)
N1—C7—C8—C9	−0.5 (3)	C16—C17—C18—C19	0.3 (4)
N2—C7—C8—C9	177.44 (19)	C17—C18—C19—C20	−2.1 (4)
C13—C8—C9—C10	1.4 (3)	C18—C19—C20—C15	0.9 (4)
C7—C8—C9—C10	−176.1 (2)	C16—C15—C20—C19	2.0 (3)
C8—C9—C10—C11	−1.2 (3)	C14—C15—C20—C19	179.9 (2)
C9—C10—C11—C12	0.0 (4)		

### Hydrogen-bond geometry (Å, °)

**Table:**

*D*—H···*A*	*D*—H	H···*A*	*D*···*A*	*D*—H···*A*
N3—H3a···N4^i^	0.93 (2)	2.13 (2)	3.058 (3)	174.5 (18)
N4—H4a···O1^ii^	0.95 (2)	2.00 (3)	2.946 (3)	174.4 (19)
N4—H4b···N3	0.86 (2)	2.35 (2)	3.006 (3)	133.8 (19)
O1—H1···N1	0.95 (3)	1.88 (3)	2.814 (2)	167 (3)
C14—H14···Cg^i^	1.00	2.44	3.408 (2)	162

Symmetry codes: (i) *x*, *y*−1, *z*; (ii) *x*+1, *y*, *z*.
